# Supplementary material for: Binary matrix factorization on special purpose hardware
Source: PLoS One. 2021 Dec 16;16(12):e0261250. doi: 10.1371/journal.pone.0261250 (PMC8675762; doi:10.1371/journal.pone.0261250)
Supplement: S1 Text — Contains supporting text to the main manuscript. (ZIP) [file pone.0261250.s001.zip › S1_Text.pdf]

Binary matrix factorization on special purpose hardware: Supplementary material

Osman Asif Malik*† Hayato Ushijima-Mwesigwa‡ Arnab Roy‡ Avradip Mandal‡
Indradeep Ghosh‡

Implementation of thresholding method for BMF

Zhang et al. [4, 3] present two algorithms for BMF. The penalty based version (Algorithm 1 in [3]) is available in existing Python implementations; see e.g. [1] and [2]. We have not been able to find an implementation of their thresholding algorithm (Algorithm 2 in [3]). For their thresholding algorithm, they give two alternative solution approaches: Discretization and gradient descent. They only use the gradient descent based variant in their experiments, since they say discretization is too computationally expensive. However, as we show below, if the discretized variant is implemented carefully, it can find the optimal solution to the thresholding approach in $O(mnr^2 \min(m,n))$ time, which is fast enough for our experiments.

For a matrix $\boldsymbol{W} \in \mathbb{R}^{m \times r}$, we define g to be the function that outputs a vector $\boldsymbol{v} = g(\boldsymbol{W}) \in \mathbb{R}^{mr}$ such that $\boldsymbol{v}$ contains all the elements in $\boldsymbol{W}$, ordered in descending order, i.e., such that $v_i \geq v_j$ whenever $i < j$. When two entries in $\boldsymbol{W}$ have the same value, which of them comes first in $\boldsymbol{v}$ does not matter, as long as this is decided in some consistent fashion. We also assume that the functions f_r and f_c take an entry v_k as input and return the row and column position, respectively, of this entry in $\boldsymbol{W}$. For example, if the number v_k corresponds to an entry in position (i,j) in $\boldsymbol{W}$, then $f_r(v_k) = i$ and $f_c(v_k) = j$. The thresholding function $\theta : \mathbb{R} \to \{0,1\}$ is defined as

$$\theta(x) \stackrel{\text{def}}{=} \begin{cases} 1 & \text{if } x \geq 0, \\ 0 & \text{if } x < 0. \end{cases}$$

The number η is any positive constant. Algorithm 1 provides our implementation of the thresholding method by [3]. We use a variant of it coded in Python in our experiments.

The reason this algorithm works is that there are only $mr+1$ ways to threshold $\boldsymbol{W}$ and only $nr+1$ ways to threshold $\boldsymbol{H}$. The nested for loops search through all the thresholdings that result in nonzero values of $\boldsymbol{X}$. Moreover, since each entry of $\boldsymbol{X}$ is nondecreasing during the iterations of the inner for loop, if $\boldsymbol{X}$ is nonbinary for some p and some $q = k_i$, it will be nonbinary for that same p and all subsequent $q = k_j$ where $j > i$. And this is why it is fine to break out of the inner for loop, since none of the following iterates are going to lead to valid factorizations. Algorithm 1 is presented at a higher level to make it easier to follow. We now discuss some implementation details that make the algorithm faster and allow us to achieve $O(mnr^2 \min(m,n))$ run time.

- Line 7: Setting $\boldsymbol{W}' = \boldsymbol{0}_{m \times r}$ before the outer for loop, it is sufficient to make the update $w'_{f_r(p) f_c(p)} = 1$ on this line.
- Line 9: Setting $\boldsymbol{H}' = \boldsymbol{0}_{r \times n}$ before the inner for loop, it is sufficient to make the update $h'_{f_r(q) f_c(q)} = 1$ on this line.

*University of Colorado Boulder, osman.malik@colorado.edu
†Work done while at Fujitsu Research of America, Inc. USA
‡Fujitsu Research of America, Inc. USA, hayato@fujitsu.com, {aroy,amandal,ighosh}@us.fujitsu.com

Algorithm 1: Efficient implementation of Algorithm 2 in [4]

Data: Matrices $\boldsymbol{A}$, $\boldsymbol{W}$, $\boldsymbol{H}$ such that $\boldsymbol{A} \approx \boldsymbol{W}\boldsymbol{H}$ is a nonnegative matrix factorization
Result: Binary matrices $\widetilde{\boldsymbol{W}}$, $\widetilde{\boldsymbol{H}}$ such that $\boldsymbol{A} \approx \widetilde{\boldsymbol{W}}\widetilde{\boldsymbol{H}}$ is a binary matrix factorization

`/* Initialization */`
1 $\widetilde{\varepsilon} = \|\boldsymbol{A}\|_{\mathrm{F}}^2$
2 $\widetilde{\boldsymbol{W}} = \boldsymbol{0}_{m \times r}$
3 $\widetilde{\boldsymbol{H}} = \boldsymbol{0}_{r \times n}$
4 $\boldsymbol{v} = g(\boldsymbol{W})$
5 $\boldsymbol{k} = g(\boldsymbol{H})$

`/* Search through grid */`
6 **for** $p = v_2, \dots, v_{mr}, v_{mr} + \eta$ **do**
7     $\boldsymbol{W}' = \theta(\boldsymbol{W} - p)$
8   **for** $q = k_2, \dots, k_{nr}, k_{nr} + \eta$ **do**
9         $\boldsymbol{H}' = \theta(\boldsymbol{H} - q)$
10         $\boldsymbol{X} = \boldsymbol{W}'\boldsymbol{H}'$
11         $\varepsilon = \|\boldsymbol{A} - \boldsymbol{X}\|_{\mathrm{F}}^2$
12   Set flag to true if $\boldsymbol{X} \in \{0, 1\}^{m \times n}$, false otherwise
13   **if** *flag is false* **then**
14   break `// Since` $\boldsymbol{X}$ `won't be binary for subsequent` q`s`
15   **else if** $\varepsilon < \widetilde{\varepsilon}$ **then**
16             $\widetilde{\boldsymbol{W}} = \boldsymbol{W}'$
17             $\widetilde{\boldsymbol{H}} = \boldsymbol{H}'$
18             $\widetilde{\varepsilon} = \varepsilon$
19   **end**
20   **end**
21 **end**
22 **return** $\widetilde{\boldsymbol{W}}$ and $\widetilde{\boldsymbol{H}}$

- Line 10: Setting $\boldsymbol{X} = \boldsymbol{0}_{m \times n}$ before the inner for loop, it is sufficient to make the update $\boldsymbol{x}_{*f_c(q)} = \boldsymbol{x}_{*f_c(q)} + \boldsymbol{w}'_{*f_r(q)}$ on this line.
- Line 11: Assuming that the column $\boldsymbol{x}_{*f_c(q)}$ was saved prior to making the update on line 10 in a vector $\boldsymbol{t}$, we can now compute
$$\varepsilon = \varepsilon + \|\boldsymbol{a}_{*f_c(q)} - \boldsymbol{x}_{*f_c(q)}\|_{\mathrm{F}}^2 - \|\boldsymbol{a}_{*f_c(q)} - \boldsymbol{t}\|_{\mathrm{F}}^2.$$
- Line 12: The value of the flag can be computed by evaluating the truth of $[\boldsymbol{x}_{*f_c(q)} \in \{0, 1\}^m]$.
- Lines 16 and 17: Instead of updating all entries in the two matrices, it is sufficient to just keep track of the pair (i, j) corresponding to the $p = v_i$ and $q = k_j$ that resulted in the new best decomposition. The matrices $\widetilde{\boldsymbol{W}}$ and $\widetilde{\boldsymbol{H}}$ to be returned can then be computed right before the return statement.

Assume $m \leq n$. The complexity of each inner loop iteration in the algorithm is $O(m)$. This is then repeated $O(mnr^2)$ times over all inner and outer loop iterations. The total cost is therefore $O(mnr^2 \cdot m)$. If $n \leq m$, we can transpose the input matrix before applying the algorithm, resulting in a complexity of $O(mnr^2 \cdot n)$. The complexity is therefore $O(mnr^2 \min(m, n))$, as claimed.

Details on baseline method

Algorithm 2 describes our baseline method in detail. On lines 1 and 2, $\boldsymbol{U}$ and $\boldsymbol{V}$ are initialized to zero matrices. On line 4, the current residual $\boldsymbol{E}$ is computed. On line 5, the least index i' is computed such that the sum of the i'th row of $\boldsymbol{E}$ is equal to the max row sum of $\boldsymbol{E}$. A similar index is computed for the columns of the residual on line 6. The update on lines 8 and 9 eliminate the densest column in the residual. Similarly, the update on lines 11 and 12 eliminate the densest row in the residual. The first if statement determines if a row or column should be eliminated in order to maximizing the reduction in the residual. Finally, the second if statement checks if a simple rank-1 approximation consisting of only ones yields a better approximation than the row/column elimination procedure.

Algorithm 2: Baseline method

Data: Matrix $\boldsymbol{A}$, target rank r
Result: Binary matrices $\boldsymbol{U}, \boldsymbol{V}$ such that $\boldsymbol{A} \approx \boldsymbol{U}\boldsymbol{V}^\top$ is a binary matrix factorization
1 Set $\boldsymbol{U} = \mathbf{0}_{m \times r}$
2 Set $\boldsymbol{V} = \mathbf{0}_{n \times r}$
3 **for** $k \in [r]$ **do**
4   Set $\boldsymbol{E} = \boldsymbol{A} - \boldsymbol{U}\boldsymbol{V}^\top$ `// Compute residual`
5   Let $i' = \min\{i \in [m] \ : \ \sum_j e_{ij} = \max_\ell \sum_j e_{\ell j}\}$
6   Let $j' = \min\{j \in [n] \ : \ \sum_i e_{ij} = \max_\ell \sum_i e_{i\ell}\}$
    `/* Eliminate row/column in residual with most nonzeros */`
7   **if** $\sum_j e_{i'j} < \sum_i e_{ij'}$ **then**
8   Set $\boldsymbol{u}_{*k} = \boldsymbol{e}_{*j'}$
9   Set $v_{j'k} = 1$
10   **else**
11   Set $u_{i'k} = 1$
12   Set $\boldsymbol{v}_{*k} = \boldsymbol{e}_{i'*}$
13   **end**
14 **end**
`/* Check if trivial rank-1 approximation of all 1's is better */`
15 **if** $\|\boldsymbol{A} - \mathbf{1}_{m \times n}\|_{\mathrm{F}}^2 < \|\boldsymbol{A} - \boldsymbol{U}\boldsymbol{V}^\top\|_F^2$ **then**
16   Set $\boldsymbol{U} = [\mathbf{1}_{m \times 1} \ , \ \mathbf{0}_{m \times (r-1)}]$
17   Set $\boldsymbol{V} = [\mathbf{1}_{n \times 1} \ , \ \mathbf{0}_{n \times (r-1)}]$
18 **end**
19 **return** $\boldsymbol{U}, \boldsymbol{V}$

Algorithm for generating binary matrices

In this section, we present the algorithm we use for generating $\boldsymbol{A}$ with an exact rank-r decomposition in the synthetic experiments. It is given in Algorithm 3. The factor matrices $\boldsymbol{U}$ and $\boldsymbol{V}$ are randomly initialized with entries drawn independently from Bernoulli distributions with success probabilities p_U and p_V, respectively. We use $p_U = p_V = 0.7$ in our experiments. At this point, if $r > 1$, it may be the case that the product $\boldsymbol{A} = \boldsymbol{U}\boldsymbol{V}^\top$ is not binary. The purpose of the while loop is to eliminate nonzero entries in $\boldsymbol{U}$ and $\boldsymbol{V}$ until $\boldsymbol{U}\boldsymbol{V}^\top$ is binary. ρ is a function which returns the average of the entries in a matrix, e.g., $\rho(\boldsymbol{U}) = \sum_{ik} u_{ik}/(nr)$. For each iteration of the while loop, a nonzero entry is eliminated in the factor matrix with the highest entry average. Eventually, $\boldsymbol{A} = \boldsymbol{U}\boldsymbol{V}^\top$ will be binary, at which point the while loop terminates and $\boldsymbol{A}$ is returned.

Algorithm 3: Algorithm for generating binary matrix which has an exact rank-r BMF

Data: Matrix dimensions m, n, rank r, target initial densities $p_U, p_V \in (0, 1)$
Result: Binary matrix $\boldsymbol{A}$ which has an exact rank-r BMF

```
Draw all entries u_ik ~ Bernoulli(p_U) and v_jk ~ Bernoulli(p_V) independently
Set A = UV^⊤
while A has an entry exceeding 1 do
    if ρ(U) ≥ ρ(V) then
        Let φ = {i ∈ [m] : ∃j ∈ [n] satisfying a_ij > 1 and Σ_k u_ik > 2}
        Draw index i' ∈ φ uniformly at random
        Choose nonzero entry in the row u_{i'*} uniformly at random and set it to 0
    else
        Let φ = {j ∈ [n] : ∃i ∈ [m] satisfying a_ij > 1 and Σ_k v_jk > 2}
        Draw index j' ∈ φ uniformly at random
        Choose nonzero entry in the row v_{j'*} uniformly at random and set it to 0
    end
    Set A = UV^⊤
end
return A
```

References

[1] Nimfa python library, 2016. URL `http://nimfa.biolab.si/`.

[2] Christopher Schinnerl. PyMF - python matrix factorization module, 2017. URL `https://github.com/ChrisSchinnerl/pymf3`.

[3] Zhong-Yuan Zhang, Tao Li, Chris Ding, Xian-Wen Ren, and Xiang-Sun Zhang. Binary matrix factorization for analyzing gene expression data. *Data Mining and Knowledge Discovery*, 20(1):28, 2010.

[4] Zhongyuan Zhang, Tao Li, Chris Ding, and Xiangsun Zhang. Binary matrix factorization with applications. In *Seventh IEEE International Conference on Data Mining (ICDM 2007)*, pages 391–400. IEEE, 2007.
