# Supplementary material for: Binary matrix factorization on special purpose hardware
Source: PLoS One. 2021 Dec 16;16(12):e0261250. doi: 10.1371/journal.pone.0261250 (PMC8675762; doi:10.1371/journal.pone.0261250)
Supplement: Attachment — Submitted filename: Response to Reviewers.pdf [file pone.0261250.s002.pdf]

Applied Math
UNIVERSITY OF COLORADO **BOULDER**

Dept. of Applied Mathematics
Engineering Center, ECOT 225
526 UCB
Boulder, Colorado 80309-0526

Osman Asif Malik
t 619 452 5344
osman.malik@colorado.edu

July 10, 2021
Re: Response to Reviewers

We thank the academic editor and the reviewers for their time and effort spent reviewing our manuscript. We greatly appreciate the thoughtful comments and suggestions. We address each of the comments by the academic editor and the reviewers below.

<u>Academic editor</u>

1. We went through the provided guides and modified our manuscript to ensure that it follows the guidelines. Among other things, we removed the heading numbers and fixed all section references so that they use \nameref instead of \ref, we moved the supplementary material to a separate document, and we added a section on supporting information in the main manuscript.

2. We provide updated Funding and Competing Interests statements in the cover letter. We also ensured that the author roles were accurate when submitting this revised manuscript.

3. We have added a supporting information section to the main manuscript and updated all internal references accordingly.

We checked our figures using PACE. Figs 2 and 3 in our original manuscript (Figs 1 and 2, respectively, in revised manuscript) passed the test fine. Fig 1 in our original manuscript didn't pass the test, so we replaced the figure with an example instead (see Example 1 in the revised manuscript) which shows the same exact BMF as the figure did.

<u>Reviewer 1</u>

Responses to comments in Question 5 follow below.

Comment: "*The authors should define all the notations and acronyms before using them.*"
Response: We have gone through the manuscript and made sure all acronyms and abbreviations (except some very well known and widely used ones like "NP-hard") are defined when first used.

Comment: "*BLS problems can be solved via a QUBO formulation can be explained in two ot three lines.*"
Response: In the section "Handling large rectangular matrices" below Eq (19) in the updated manuscript, we have added two sentences which explain how the BLS problem can be formulated as a QUBO problem. We also point to the relevant references where this formulation was first proposed.

Comment: "*Add the advantages of the proposed system in one quoted line for justifying the proposed approach in the Introduction section.*"
Response: We added a sentence in the 4th point in the list of contributions (right above the section "Binary matrix factorization") which more clearly states what the advantage of our method is over those by Zhang et al.

Comment: "*The motivation for the present research would be clearer, by providing a more direct link between the importance of choosing your own method.*"
Response: We added a couple of additional sentences in the introduction that explain how our work fits into the bigger picture of investigating post-Moore's law technologies, and how this is particularly important for data mining primitives like binary matrix factorization.

Reviewer 2

Question 3 regarding data availability: All our real datasets are publicly available. In the initial submission, we provided links to the cancer datasets. In this revised submission, we have added the url for each the cancer and MNIST datasets directly in the main text rather than the bibliography to make it easier for readers to quickly find the data.

Response to comments in Question 5 follow below.

Comment: "*1. Author failed to highlight contributions in this article.*"
Response: Note that our original submission did contain a list of contributions in the introduction. In this revised submission, we have expanded the first bullet point so that it now also mentions the baseline method. We have also expanded the fourth bullet point so that it now mentions the key benefit of our method in the experiments. Finally, we also added two sentences in the introduction which helps clarify our contribution by putting it into a broader context.

Comment: "*2. How is the paper different from existing works? What gaps they identified in existing works? How the proposed approach solves the research/scientific problems identified in the existing works? Compare the results with recent state of the art.*"
Response: Broadly, the point of our paper is to investigate how binary matrix factorization can be done on post-Moore's law hardware, more specifically, hardware based on solving QUBO problems. We have added comments on this in the introduction. In order to clarify how our paper differs from previous works that use special purpose / post-Moore's law hardware to solve linear algebra tasks, we have expanded the paragraph that deals with this in the section "Related work". In particular, we now clearly point out what is different in our paper/method compared to the previous works mentioned.

Comment: "*3. Authors are used baseline method but there is no discussion about this abstract, introduction etc.*"
Response: We have added comments on the baseline method in the abstract and introduction.

Comment: "*4. In discussion section authors stated that "Our DA+ALS BMF has the same or better accuracy as 333 either of the methods by [2, 3] in 72 of the 75 experiments reported in the tables in this paper, and a strictly better accuracy in 57 of the experiments". But I can't find any table that reported these experiments.*"
Response: Table 1 contains 15 experiments: It shows results for 3 values of m, and for each m there are results for 5 different ranks, hence there are 3*5=15 experiments.

Similarly, Table 2 contains 15 experiments, Table 3 contains 15 experiments, Table 4 contains 15 experiments, Table 5 contains 5 experiments, and Table 6 contains 10 experiments.

In total, there therefore are 15+15+15+15+5+10=75 experiments.

Counting in how many of these 75 experiments DA+ALS BMF outperforms both methods by Zhang et al., we find that we have the same or better accuracy in 72 of the experiments, and strictly better accuracy in 57 of the experiments.

Comment: "*5. Improve introduction and related work sections for readability purpose.*"
Response: As discussed above, we have made updates to the abstract, introduction and related work. We hope these updates improve readability.

If any readability issues remain, please be specific when pointing them out so that we can address them.

Comment: "*6. Motivation and contributions must well written in introduction section.*"
Response: As discussed above, we have updated the list of contributions and also better motivated our work in the introduction.
